# Apologizing Again

**Published:** 1886-11

**Authors:** 


### APOLOGIZING AGAIN.

This is a fifty-six page journal, but so much matter of import to dentists has been offered us that for a number of months past each issue has been enlarged, and yet a number of accepted contributions are awaiting space. The August number contained sixty-four pages of reading matter; the September number had seventy-two, the October number sixty-four, and this is the same size. A number of reports of dental societies are in our hands waiting for room, while some exceedingly valuable articles have been necessarily withheld. The editor cannot compass impossibilities, and can but promise that as fast as possible the favors of the excellent correspondents who have sent to him contributions, shall have the place to which they are entitled.

